# Potential of the Nano-Encapsulation of Antioxidant Molecules in Wound Healing Applications: An Innovative Strategy to Enhance the Bio-Profile

**DOI:** 10.3390/molecules30030641

**Published:** 2025-01-31

**Authors:** Supandeep Singh Hallan, Francesca Ferrara, Rita Cortesi, Maddalena Sguizzato

**Affiliations:** 1 Department of Pharmaceutical Sciences and Natural Products, Central University of Punjab, Bathinda 151401, India; 2 Department of Chemical, Pharmaceutical and Agricultural Sciences, University of Ferrara, I-44121 Ferrara, Italy; frrfnc3@unife.it (F.F.); sgzmdl@unife.it (M.S.); 3 Biotechnology Interuniversity Consortium (C.I.B.), Ferrara Section, University of Ferrara, I-44121 Ferrara, Italy

**Keywords:** colloidal carriers, lipid-based nanosystems, solid lipid nanoparticles, nanostructured lipid carriers, liposomes, ethosomes, drug delivery systems, skin absorption, molecular dynamics, small angle X-Ray scattering

## Abstract

Naturally available antioxidants offer remarkable medicinal applications in wound healing. However, the encapsulation of these phytoactive moieties into suitable nano-scale drug delivery systems has always been challenging due to their inherent characteristics, such as low molecular weight, poor aqueous solubility, and inadequate skin permeability. Here, we provide a systematic review focusing on the major obstacles hindering the development of various lipid and polymer-based drug transporters to carry these cargos to the targeted site. Additionally, this review covers the possibility of combining the effects of a polymer and a lipid within one system, which could increase the skin permeability threshold. Moreover, the lack of suitable physical characterization techniques and the challenges associated with scaling up the progression of these nano-carriers limit their utility in biomedical applications. In this context, consistent progressive approaches for addressing these shortcomings are introduced, and their prospects are discussed in detail.

## 1. Introduction

Diabetic foot, venous ulcers, and pressure sores are all factors that contribute to the loss of skin integrity and lead to the formation of lesions. Specifically, a wound is a type of bleeding that penetrates the dermis layer due to trauma and has an extremely complicated healing process that involves four overlying phases, namely, hemostasis, inflammation, proliferation, and remodeling [1]. Nearly 15% of the 150 million diabetes patients globally experience diabetic wound damage, which is responsible for 50% of the cases requiring amputation therapy, with no healing improvement observed for at least up to five months of the treatment [2,3,4]. Moreover, ineffective treatment imposes a heavy economic burden on healthcare systems worldwide. The cost of wound treatment is anticipated to increase globally to USD 18.7 billion by 2027 [5,6].

In chronic hypoglycemic patients, the oxidation of glucose and the glycation of non-enzymatic proteins result in excessive Reacting Oxygen Species (ROS) generation, which ultimately enhances the production of certain enzymes, namely catalase, superoxide dismutase (SOD), and glutathione peroxidase (GPx) [7]. Unlike normal wounds, diabetic wounds are characterized by the presence of a larger number of macrophages with a higher concentration of pro-inflammatory cytokines, such as Tumor Necrosis Factor (TNF) and interleukin 6 (IL-6), as well as prolonged ROS-induced inflammation. This situation results in the inadequate apoptosis of fibroblast and poor angiogenesis and collagen deposition, leading to a delayed wound closure process [8,9]. Thus, chronic inflammation in the diabetic wound area generally leads to insufficient and deferred wound healing progress.

The skin is the largest organ, which plays a crucial role in protection from the external environment and is considered the first line of defense in the human body. The *stratum corneum* (SC), the skin’s outermost barrier, is firmly linked and serves as the primary barrier. In the SC barrier, the lipid matrix and corneocytes together form a very selective thin layer that is able to avoid/control the passage of external bodies, thus acting as the rate-limiting step for drug molecule penetration into the deeper layers of the skin. However, lipophilic compounds can easily penetrate the skin due to their higher affinity for these lipid-based tight junctions [10,11].

The free radical quenching mechanism could be helpful not only in the regulation of the pH of a wound but also in the reduction of an inflammation threshold by interrupting cellular and humoral responses. In this regard, naturally occurring antioxidant molecules have gained considerable attention, not only due to their ability to promote skin health by counteracting oxidative stress and inflammation but also for their protective effects against microbes. These plant-derived moieties have been considered safe for biomedical applications [12].

In recent investigations, several physical and chemical techniques have been utilized to boost the penetration rate of a variety of drug molecules. Some of these techniques, including iontophoresis, microneedles, and chemical penetration enhancers, are being explored by researchers. These physicochemical techniques may, however, have drawbacks, such as low stability, complex preparation methods, and mild skin irritation. Despite the efforts made to improve penetration, only a small number of bio-active drugs are now delivered via the transdermal route [13].

This review highlights the key points supporting the potential transition from the lab scale to the commercial success of colloidal nanocarriers, namely, lipid nanoparticles/vesicles, polymeric micelles, and polymer–lipid hybrid membranes and polymeric systems fulfilling the objective of wound healing.

## 2. Role of the Lipid in the Skin Permeation

Lipids are well-known biological molecules that constitute elements in the formation of plasma membranes. They are typically soluble in organic solvents and contain hydrocarbons in the small lipophilic or amphiphilic structures, playing a crucial role in energy storage. Consequently, they are highly useful in the fields of nanoscale drug delivery, food, and cosmetics [14]. The involvement of lipids in cosmetic products has been increased due to **(i)** their close structural resemblance to the lipid-constituting epidermis and SC and **(ii)** their ability to enhance skin permeation by interacting with intracellular lipid domain of corneocytes via mechanisms such as loosening, modifying polarity, fluidization, and switching [15,16]. Interestingly, the impact of lipid-based nanosystems on the structural organization of the skin can be confirmed by certain analytical techniques. For instance, the Fourier-transform infrared spectroscopy (FTIR) technique is useful for detecting endothermic transitions that indicate a transformation from a lamellar to a disordered state, while Differential Scanning Calorimetry (DSC) can assess the variations in the enthalpy of the SC lipid structures upon the application of nanoscale lipid-based structures [17,18].

Furthermore, deviations in skin impedance before and after the application of lipid vesicles have been recorded using an amperometric approach. In addition, it has been investigated that skin impedance is directly associated with skin integrity itself. A high value of skin impedance reflects the tightness of the skin barrier, indicating more intact skin, while low impedance can be recorded due to damaged SC. Progressing forward, lipid-based vesicles/particles have been applied to the excised pork skin membrane, where it was found that the vesicles were able to interact closely and blend with skin lipids, ultimately restricting the movement of ions/solutes across the SC. Remarkably, this entire process was found to be reversible. Therefore, taken together, lipid vesicles/nanoparticles (NPs) can be applied multiple times without affecting skin integrity and are thus highly compatible and suitable for transdermal drug delivery applications [19].

### 2.1. Lipid Nanoparticles (L-NPs)

The twenty percent water content retained in the SC of healthy and intact skin hampers the percutaneous uptake of exogenous moieties. Interestingly, this interruption can be resolved by inducing the occlusion effect by employing crystalline lipids with low melting points in the form of particles with ultra-small diameters. The NPs derived from these biocompatible lipids are considered safe to include in cosmetics and have garnered significant attention [20]. Lipid nanoparticles are broadly classified into two categories, namely, solid lipid nanoparticles (SLNs) and nanostructured lipid carriers (NLCs).

#### 2.1.1. Solid Lipid Nanoparticles

SLNs are colloidal carriers consisting of solid lipids stabilized by a surfactant and carrying an active molecule (e.g., a drug) dissolved or dispersed within a solid core. In particular, the biodegradable solid lipid core of SLNs is stabilized by a shell of surfactants/emulsifiers (e.g., phospholipids, poloxamers, etc.) that interacts with the fatty matrix [21,22,23]. These promising drug delivery systems are usually produced with biocompatible lipids (such as triglycerides) that remain solid at room and body temperatures. Despite their heterogeneous composition, SLNs are well tolerated by the body and are considered to be of low toxicity [24]. Furthermore, other advantages of SLNs, in addition to biocompatibility, should be mentioned, such as biodegradability, the ability to modify the physicochemical properties of drugs, ease of use, low cost, processing without organic solvents, good stability and good release characteristics [25].

Several production methods of SLNs are described in the literature [26], but the main approaches used are cold and hot homogenization [27]. However, SLNs can also be obtained from colloidal emulsions by replacing liquid lipids with solid lipid matrices at body temperature. The solid core of the L-NPs can avoid the free movement of active cargo, thereby providing mechanical and electro-chemical strength to the colloidal systems. In this way, the overall physical and chemical stability of the drug molecule can be improved with encapsulation into the SLNs [28].

SLNs undergo adhesion to the SC and induce the occlusive effect by promoting skin hydration, ultimately preventing the loss of water through evaporation [29]. Concerning the release of drugs entrapped in SLNs, it has been found that SLNs cannot penetrate the SC; however, their penetration across the hair follicles is feasible. The possible reason is that the presence of lipids, waxes, and squalene in the follicle facilitates the deeper transport of the active substance carrying L-NPs [30].

SLNs can provide long-term stability to the encapsulated molecules against environmental degradation and the coalescence of particles; they are capable of embedding a wide range of hydrophilic and lipophilic active moieties, irrespective of their molecular weights [31,32]

While designing the SLNs, pre-screening of the lipids can be helpful to examine their suitability concerning the molecules of interest to be encapsulated. Moreover, this also makes it possible to design a solo system for the delivery of several natural antioxidant molecules together. Recently, a study was conducted in which nearly fifteen lipids were screened to hold the simultaneous solubilization of three molecules (namely, epigallocatechin gallate, resveratrol, and myricetin), among which only two lipids (i.e., Gelucire^®^ 50/13 and Compritol^®^ HD5 ATO) were able to completely dissolve and host the three considered antioxidant molecules, allowing for the development of a formula for cosmetic applications [33]. It should be noted that not all types of lipids are suitable for a wide range of active cargo and should be pre-screened before converting them into the final DDS [33,34,35].

SLNs have also been ascertained as a link between traditional knowledge and modern technology. A study on the management of wound healing was conducted to encapsulate Berberis extract in a SLN-based gelling system using Compritol^®^ 888 ATO as the lipid component, due to its higher solubilization properties compared to other lipids, along with polyethylene glycol 400 (PEG 400), which was used as a cosolvent and surfactant. Hot high-pressure homogenization was used as the manufacturing technique. Under these conditions, SLNs not only improved the solubility and loading of the natural extract but also managed to accelerate wound healing without leaving any visible scars in the excision-wound model [36].

Similarly, a study aimed to develop SLNs with a high curcumin load (about 15% lipid matrix) for wound healing; they described their preparation through hot high-pressure homogenization while avoiding the use of organic solvents. The obtained SLNs showed a high drug content and solubility, high safety, and photo stability and achieved zero-order controlled release of curcumin at the wound site, allowing for a significant increase in antibacterial activity. The authors have claimed that this is the highest curcumin payload ever achieved without the use of any organic solvent [37].

**Figure 1:**
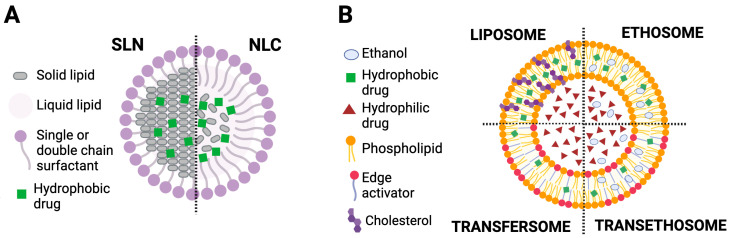
Scheme illustrating lipid-based nanoparticles (i.e., SLN and NLC) (**A**) and vesicles (i.e., liposomes, transfersomes, ethosomes, and transethosomes) (**B**). Original figure created with BioRender.com.

Although L-NPs are very efficient in protecting drug molecules from degradation, they experience low drug encapsulation, especially in the case of SLNs. A contributing factor could be the highly ordered crystalline organization formed during the cooling of the SLNs, which sometimes results in inappropriate drug release in a controlled manner [38,39]. In this context, NLCs have been considered as second-generation L-NPs. The structural features of SLNs and NLCs are illustrated in Figure 1A.

#### 2.1.2. Nanostructured Lipid Carriers

Nanostructured lipid carriers (NLCs) are considered the second-generation products of SLNs, as they are nanosized colloidal drug delivery systems whose core is made of a mixture of solid and liquid lipids [40,41]. NLCs, composed of a blend of solid and liquid lipids, are fabricated to improve the low aqueous solubility of certain antioxidant molecules. The nature of the lipid matrix allows for increased drug solubility and permeability by encapsulating the drug in a lipid shell, making NLCs an ideal carrier for drug delivery. Drug expulsion, which is a significant concern in the case of SLNs, can be avoided using NLCs. Like SLNs, NLCs can be prepared using various methods, including high-pressure homogenization, microemulsion, probe sonication, solvent diffusion, solvent emulsification evaporation, solvent injection/solvent displacement, and phase inversion [42]. They can easily solubilize the poorly soluble active molecules with extremely low permeability [43]. For example, an attempt has been made to overcome the drawback related to the solubility of the ellagic acid, where the type of the liquid lipid selected clearly improved the stability of the ellagic acid by almost two months in the case of tristearin/tricaprylin and 40 days in the case of tristearin/labrasol compared with the plain drug solution. Moreover, NLCs managed to retain 60% of the antioxidant capacity of loaded ellagic acid during the entire storage time frame [44].

These lipid nanoparticles represent biocompatible, non-toxic, and safe drug delivery systems [27,45]. Furthermore, the surface modification of NLCs using various methods can influence and modify drug targeting and increase drug residence time. Taken together, the overall effect of different lipid compositions on the encapsulation efficiency and other possible outcomes are summarized in Table 1.

It is crucial to mention here that the supremacy of NPs has so far been investigated only for one parameter at a time. For instance, most studies focus on a single aspect, such as improving the chemical stability or solubility of the active molecule or enhancing penetration efficacy across the various skin barriers, and significant data are available that evaluate multiple aspects together in a single report. Emphasizing this gap, a recent study by Wiemann and co-authors has been published, in which the L-NPs have been compared in terms of overall efficacy with other classical formulations (drug-containing oil, emulsion, creams) while keeping lipids and emulsifiers identical. The results of the study revealed that the L-NPs were able to provide an invisible patch with the highest skin hydration among the systems tested. In addition, L-NPs successfully improved the permeation of the lipophilic active substance and reflected all the advantages, thus presenting a realistic approach [55].

### 2.2. Lipid-Based Deformable Vesicles

Conventional liposomes are self-assembled vesicles based on (phospho)lipids that form a sole bilayer (unilamellar) and/or a concentric array of multiple bilayers (multilamellar) surrounding an aqueous or buffered compartment. Typically, the thickness of the phospholipid bilayer is about 4–5 nm [56], while their dimensions are generally between 30 nm and a few micrometers. The so-called “liposomology” emerged in the mid-1960s [57], and these vesicles have been widely studied for the delivery of small molecules, such as drugs and imaging agents, and biomolecules (i.e., proteins, nucleic acids) [58,59,60]. Liposomes have been proposed for different administration routes (e.g., injection, cutaneous, ocular, nasal, pulmonary), and good results have been obtained in increasing therapeutic efficacy and compliance [61].

Conventional liposomes have some limitations, such as the poor encapsulation efficiency of hydrophilic drugs, membrane instability responsible for permeability variations, and reduction of the half-life [62,63]. For these reasons, liposomology has investigated the structure of different types of vesicles, such as chitosomes, sphingosomes, niosomes, bilosomes, transfersomes, ethosomes, and invasomes. For example, chitosomes, i.e., liposomes coated with chitosan, modify the surface properties of vesicles, thereby improving their stability [64,65]. On the other hand, sphingosomes are liposomes made of sphingolipids and cholesterol [66]. Niosomes are made of non-ionic surfactants and cholesterol [67,68]. Furthermore, the presence of some ionic amphiphiles, such as dicetyl phosphate (negatively charged) or stearylamine (positively charged), are used in niosomes to improve efficacy and stability [68]. Bilosomes are vesicles made of nonionic surfactants and bile salts, and, like niosomes, are considered non-lipoid biocarriers. This type of vesicle allows for the oral administration of drugs or vaccines [69] since bile salts are endogenous surfactants widely used as absorption enhancers to improve drug permeability through biological membranes [70]. Transfersomes present in their composition, in addition to phospholipids, an edge activator (EA) (such as Tween 80, Span 80, sodium cholate), which, as a membrane softening agent, facilitates ultra-deformability [71]. Consequently, after cutaneous application, transfersomes can self-optimize their membrane flexibility and spontaneously cross the skin [72,73]. Several transfersome-based formulations are currently being evaluated in different phases of clinical studies [73,74,75]. Ethosomes are vesicles composed mainly of phospholipids when in the presence of a relatively high concentration of ethanol (20–45%), glycols, and water that rearranges to form multiple concentric layers of bilayers with high flexibility [76]. The presence of ethanol has been shown to stabilize the vesicles, allowing for the control of their entrapment efficacy and facilitating transdermal drug delivery [77,78]. Finally, invasomes are vesicles composed of phospholipids, ethanol, and terpenes that make these vesicles soft, with suitable transdermal penetration properties. Consequently, these nanovesicles have excellent capabilities of improving drug permeability in the skin and diminishing systemic circulation absorption, thus confining the activity of the transported drugs within the skin layer [79].

Furthermore, the results of liposomology investigations on the peculiar bilayer structure of these nanovesicles have allowed for the use of these vesicles in the cosmetic [80] and food [81] fields.

Liposomes are bio-inspired systems that exist in the form of lipid droplets. These lipid droplets, enclosed by phospholipid monolayers, are referred to as depots of various esterified fatty acids and sterols within eukaryotic cells. These organelles are directed toward the cytoplasmic phase of the endoplasmic reticulum membrane and are ultimately released into the cytosol [82,83]. Liposomes composed of double phospholipid membranes (single or multiple bilayers with the potential to enclose an aqueous phase), are among the few carriers that have achieved clinical and commercial success due to their flexibility in terms of physicochemical and biophysical characteristics, including a major aqueous core and lipid shell, as well as their tendency to self-assemble. All these merits enable them to attain a high percentage of encapsulation for both hydrophilic and hydrophobic active moieties.

In general, the vesicles possess a negative charge on their surface due to the presence of a phosphate group on the heads of soy lecithin. In certain cases, however, these anionic vesicles are not able to deliver therapeutic agents precisely at the cytoplasmic level due to the repulsive force exerted by the cell membrane. Notably, the charge on the surface of the vesicles can be modified via electrostatic deposition of different cationic polymers [84,85]. Successful anionic/cationic charge deployments can be confirmed by shifts in the zeta potential values from approximately 0 ± 5 mV to highly positive/negative ends. A very similar study has demonstrated the effect of strong anionic (dicetyl-phosphate) and cationic (dodecyl-dimethyl-ammonium chloride, di isobutylphenoxy ethyl dimethyl benzyl ammonium chloride, and octadecyl-amine) surfactants embedded in the vesicles, as evidenced by the deviations in zeta potential measurements [86]. This surface charge modification is very helpful in transfection and gene knockings, as it provides higher cellular uptake of the selected genetic materials.

In addition to the enhancement in the uptake at the cellular level, charged moieties can also aid in achieving other physiological targets. This can be explained further by considering the latest findings, in which liposomes have been selected for the oral delivery of natural extracts containing anthocyanin, representing a commendable approach to exploiting liposomes in food sciences research. Furthermore, the antioxidant activity was maintained in the entrapped anthocyanin molecules, along with improved overall stability. In addition, the chitosan adsorbed on the surface of the liposomes helped in attaining prolonged residence time in vivo and prolonged release of molecules within the stomach and intestine [87]. The presence of the chitosan coating prevented the direct erosion of the phospholipid layer by lipase, bile salts, gastric juice, and acids present in the stomach [87].

The ultra-deformable lipid vesicles, namely, ethosomes (ethanol + liposomes), exhibit a higher loading capacity for poorly water-soluble molecules than classical liposomes. Indeed, the presence of ethanol stabilizes the vesicles and controls their entrapment efficacy. This effect may be due to the combination of ethanol and phospholipid, which causes a noticeable rearrangement in the skin lipids [88].

The molecular effect of ethanol on the skin has been assessed via simulation studies. Ethanol is well known for enhancing the permeation of both polar and non-polar moieties and acts as a permeation enhancer [89]. However, the exact mechanism involved in this is not clearly understood; lipid extraction from the SC, fluidization of the SC lipid bilayers, alteration in configuration of SC proteins, and increased drug solubility within SC lipids are some of the proposed mechanisms [89,90]. Simulation studies have revealed that the selective fatty acids matrix present in the lipid bilayer of the skin can be compromised immediately upon exposure to ethanol. As a result, ethanol permeates deeply into the lipid bilayer and creates a passage for drug molecules and carrier systems [90]. The specific diameter range of these lipid vesicles can project different features, which are given in Table 2.

The charge on the vesicle surface is a crucial factor for transcutaneous diffusion, particularly concerning the presence of negatively charged lipids in the SC layers. For instance, positively charged liposomes can penetrate deeper and disrupt the tight junctions of the lower epidermis layers. Notably, vesicles with altered surface charges can provide a good transdermal effect. Research has been conducted to compare anionic and cationic ultra-deformable vesicles with conventional vesicles for the delivery of imperatorin (a Chinese herbal medicine). The phospholipid and cholesterol-containing vesicles have been modified with either an anionic surfactant (dicetyl phosphate) or cationic agents (octadecylamine/stearylamine). Furthermore, tween 20 has been employed as the edge activator. These positively charged vesicles, also known as transethosomes, have shown the highest encapsulation capacity, along with the highest skin penetration levels compared to other existing vesicles [98]. Furthermore, the FTIR and DSC results have supported the phenomenon of the disruption of the fluidity of SC lipids by the vesicles without undergoing any considerable adverse alterations. Indeed, Lőrincz et al. [99], studying the influence of ursolic acid on the structural and morphological characteristics of DPPC-based liposomes using different techniques, including DSC and Fourier-transform infrared spectroscopy (FTIR), found that the drug induced small and stable perturbations in the thermal behavior of the vesicular system. On the other hand, the FTIR spectroscopy results showed that the drug led to the formation of a new band around 1700 cm^−1^ [99,100]. Moreover, the group of Abboud [101] examined the effects of certain triterpenes on the fluidity of liposome membranes using DSC, Raman spectroscopy, and fluorescence anisotropy. In particular, the DSC results demonstrated that the presence of triterpenes in the vesicles was revealed by a shift towards lower values of the main phase transition temperature and the disappearance of the pretransition. Evidently, lipid-based vesicles can deliver drug molecules deeply without affecting skin integrity [96,98].

The deformability of these vesicles is a critical parameter that can be regulated by various factors, such as the concentration of the employed lipid, the methodology adopted for the fabrication of these vesicles, and the presence of a surfactant/surfactants [102]. The structural features of liposomes, transfersomes, ethosomes, and transethosomes are presented in Figure 1B.

## 3. Polymeric Nano-Transporters

**Figure 2:**
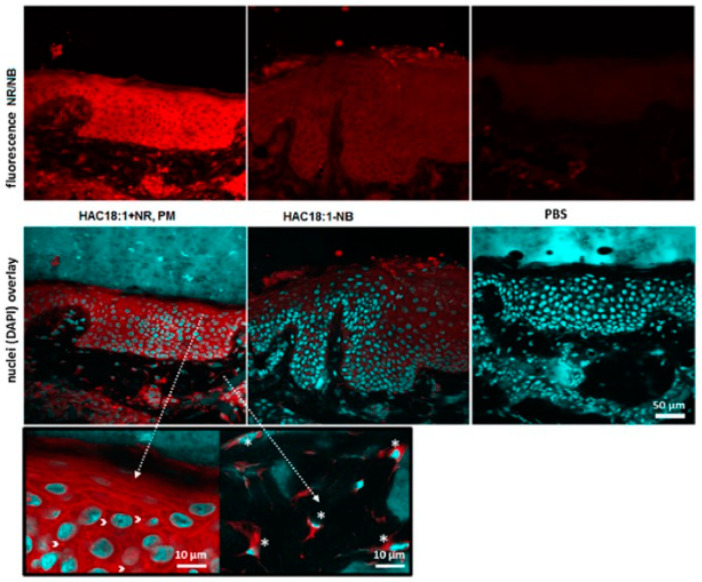
Confocal images of cross sections of porcine skin after 20 h of treatment with PBS (used as a control), hyaluronan polymeric micelles loaded with Nile Red (HAC18:1 + NR, PM), and hyaluronan polymeric micelles covalently labeled with Nile Blue (HAC18:1-NB). Both NR and NB exhibit red fluorescence. The bottom images show merged views of NB or NR fluorescence and stained cell nuclei (blue). Details (in a black frame) of the skin sample treated with NR-loaded micelles include keratinocytes with vesicles (arrows) on the left and dermal fibroblasts (*) on the right. (With Elsevier permission from reference [105], License Number 5947591285425).

In promoting non-invasive strategies, polymeric self-assembled micelles have gained significant interest in recent studies. It is already a well-known mechanism that surfactants have the ability to spontaneously self-assemble into spherical micelles after reaching concentrations above the critical micelle concentration (CMC) in aqueous media, thereby reducing surface-free energy. These polymeric micelles can encapsulate poorly water-soluble drug molecules. The employed polymers can disrupt the bilayer lipid arrangements of cells, thereby enhancing the extent of permeation of associated drugs [103]. Moreover, if the polymers are positively charged, they interact with the negative charge on the surface of the skin, improving drug diffusion to the deeper skin regions [104]. The smaller diameter of micelles also facilitates their passage across various skin barriers. Recent confocal images have shown that micelles of sodium oleyl hyaluronan (HAC18) are confined within keratinocytes and fibroblasts (Figure 2) [58]. In particular, HAC18 micelles loaded with Nile Red (HAC18-NR) and labeled with Nile Blue (HAC18-NB) were detected in the cytoplasm surrounding the cell nuclei in the epidermal and dermal layers [105].

On the other hand, the micelles remained intact up to 10 mm deep in the skin, but investigations of deeper layers exhibited slow micelle disruption [105]. This is probably due to low flexibility. It should be emphasized that they can travel intact only through hair follicles and accumulate there. In addition, the polymeric vesicles face issues regarding instability at CMC values and require complex characterization methods.

Although polymeric micelles possess some limitations, their merits cannot be overlooked. Therefore, the possibility of combining the merits of the polymer micelles and lipid vesicles can establish a synergist relationship with better efficacy in the process of wound healing [106,107]. Interestingly, the polymer–lipid hybrid vesicle system can integrate three effects together into a single approach, including the polymeric effect, ethanol effect, and ethosomal effect. The resulting vesicles are highly deformable and can facilitate drug diffusion deeper into the skin layers with better control. Unlike polymeric micelles, which mostly accumulate in hair follicles, these hybrid vesicles are particularly effective in enabling their passage across the layers of the disrupted SC [108].

**Figure 3:**
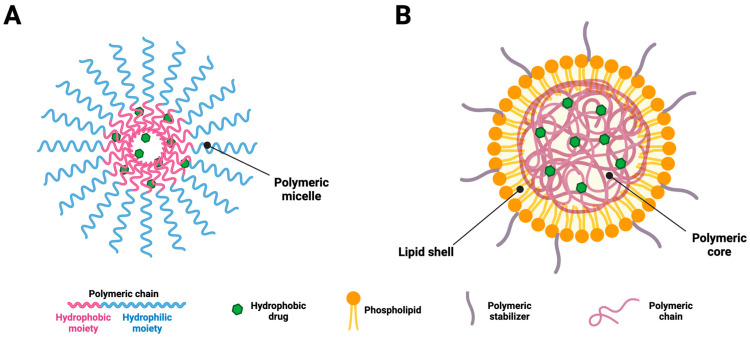
Scheme illustrating a polymeric micelle (**A**) and a lipid–polymer hybrid membrane (**B**). Original figure created with BioRender.com.

Although the use of polymers in lipid vesicles was not introduced recently [109,110,111], the polymeric arrangement within the core of lipid vesicles could open a new research horizon in the field of drug delivery. Figure 3 provides an overview of polymeric micelles and polymer–lipid hybrid membranes.

## 4. Microneedle-Assisted Transdermal Drug Delivery

The term “transdermal effect” refers to the release of a drug in the epidermal region to reach the bloodstream. Although nanosystems composed of lipids or polymers show very promising results, the microneedle approach has also demonstrated clinical success. The principle of microneedles (MNLs) relies on their ability to induce the reversible micropores across the SC due to their needle-like projections, which facilitate site-specific drug release with minimal side effects, making them preferable to hypodermic injections [112]. MNLs can overcome the significant barrier posed by the SC by piercing into it, thereby enhancing skin permeability and the flux of the molecules with high molecular size and weight [113,114].

MNLs are available in various needle lengths up to 1100 µm, depending on their intended purpose. In addition, they are highly biocompatible, as they are composed of different carbohydrates (polysaccharides), and their surface properties can also be modified. To the best of our knowledge, no in situ infections or interference with skin functionality have been recorded. Moreover, MNL devices deliver not only proteins, nucleic acids, and virus-like entities, but also vaccines and hormones [115].

The MNL-associated ferulic acid peel has been applied to various subjects aged 45–60 years to reduce signs of photo-aging. Improved skin elasticity was observed in these designed MNLs containing ferulic acid [116]. Remarkably, the MNLs are not only able to enclose the antioxidant molecules but also protect them from external degradation/oxidation. For instance, a combinational approach has been employed in the treatment of atopic dermatitis wherein the epigallocatechin gallate (a polyphenol derived from green tea) has been exploited. In addition to hydrogen atom donation, it has the ability to chelate various metal ions susceptible to oxidation. Moreover, it can interrupt degranulation and histamine release from mast cells and basophils. Despite its advantages, this molecule faces numerous challenges related to poor bioavailability. In order to enhance its stability, reducing agents, namely, poly-γ-glutamate (γ-PGA), have been employed as MNL material. Interestingly, the developed MNL has reduced the dosing frequency from once a week, and it has been found to be very effective and safe when applied in in vivo testing [117].

Moving forward, the synergistic response can be elicited by employing MNLs and nanosystems together as a single model approach. Three vesicles, namely, liposomes, transfersomes, and ethosomes, have been proposed to enclose vitamin B12 (cyanocobalamin). The movement of drug-carrying lipid vesicles across the skin has been compared both alone and with the assistance of MNLs. It has been found that the flux of entrapped molecules was enhanced when using MNL. The penetration of MNL into deeper skin layers has been further verified using methylene blue staining [118].

In addition to lipid vesicles, polymeric micelle technology was combined with MNL, where the molecule of interest, curcumin, was encapsulated in dithiodipropionic acid-oligomeric hyaluronic acid, and the MNL matrix was made by the hyaluronic acid composite. This system has managed to deliver around 75% of curcumin within a six-hour window, which is relatively fast [119].

MNLs, together with SLNs carrying highly lipophilic fluorescent probes as a payload, have been employed to track their in vivo fate. The diameter of SLNs, around 150 nm, was maintained throughout the entire storage period without any observed aggregation, sedimentation, or quenching of fluorescence with impaired signal quality. It is important to mention that the transdermal diffusion of the drug is highly dependent on the thickness of the skin and some miscellaneous factors, including skin disease, the site of MNL application, age, and gender, which should be considered and optimized at the clinical level [120].

Similarly, lipid-based nano-capsules have been investigated as a carrier for hypericin (a therapeutically active hydrophobic photosensitizer for combating skin cancer). The quantitative encapsulation efficiency and cell uptake potential have been monitored. The lipid nanocarrier implanted into MNLs not only bypassed the issues associated with hypericin (poor solubility and skin permeability), as well as the tendency to undergo aggregation upon exposure to the physiological medium, but also significantly reduced tumor size in the rat model [121].

As previously discussed, better results could be achieved by combining two approaches. This concern has been addressed by designing SLNs carrying curcumin, which are further enclosed in MNLs using the micromolding technique. The prepared MNL patch with minimal invasion has remarkably treated Parkinson’s disease. Due to the needle homogeneity, a significant neuroprotective effect (characterized by a reduced degree of bradykinesia, improved motor coordination, and balance ability) has been recorded in Swiss albino mice with Parkinson’s disease. Consequently, greater bioavailability *via* transdermal absorption could be increased by developing a microneedle patch [122].

Polymeric MNLs have been employed, along with NLCs carrying β-sitosterol, in hair loss therapy. The flux rate across the rat skin for MNL-guided NLCs was recorded as comparatively higher than the NLCs alone. These results correlated very well with hair growth. Therefore, MNL-guided nanosystems may have greater potential to treat androgenic alopecia. Moreover, chitosan was selected as a material to fabricate MNLs in order to ensure progressive disintegration without eliciting a significant immune response [123,124].

**Figure 4:**
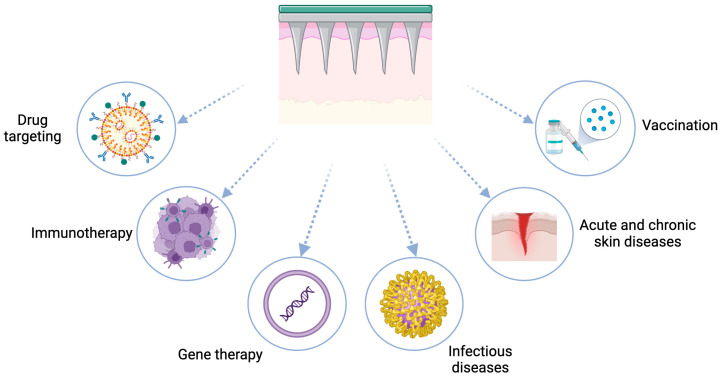
Applications of MNLs for transdermal delivery. Original figure created with BioRender.com.

Taken together, MNLs have numerous applications in various areas of biomedical sciences, including drug targeting, immunotherapy, gene therapy, and skin health maintenance, as shown in Figure 4.

However, it is very important to further investigate if MNLs are useful only in treating local skin infections or if they are also effective in transporting drugs to systemic circulation. Indeed, sometimes the drug molecules cannot infiltrate into the next layer without achieving saturation in the previous layer. Therefore, the use of a drug at higher concentrations needs to be projected accurately, which is very challenging and might give rise to serious toxicity concerns [118]. Moreover, it is imperative to emphasize that the abovementioned synergism is not obvious or easy to establish in all the cases, and its clinical and commercial success is still underway [125].

## 5. Investigation of the Structural Organization and Physical Aspects of the Lipid Nanosystems

Among the different techniques useful for investigating the structural organization of lipid-based nanosystems, the following can be mentioned.

Scanning Electron Microscopy (SEM) can provide detailed images of the surface morphology, including the shape, texture, and size of lipid- and polymer-based carriers, offering insights into their structural properties [126,127,128]. Nevertheless, SEM is not often employed in the case of lipid-based nanostructures, as this tool is destructive to the sample in nature. Recently, environmental scanning electron preferred, particularly for analyzing liposomes [129].

Transmission Electron Microscopy (TEM) delivers high-resolution images of the internal structure and size of the nanocarriers, which is crucial for evaluating encapsulation efficiency and the distribution of antioxidant molecules within the carriers [130,131,132]. The particle dimensions and morphology can be visualized using the TEM Imaging technique. The deposition of the samples is performed on the carbon film-coated copper grid. The TEM analysis procedure is very straightforward; however, it involves hot air-drying the sample before staining, which can result in shrinkage of the liposomes [84]. This pre-treatment method can be further modified. The freeze structure technique is preferable due to the vitrification of the sample via rapid freezing, which eliminates the possibility of heating the sample [129,133].

X-Ray Diffraction (XRD) is pivotal in determining whether the encapsulated antioxidants and carrier matrix are in a crystalline or amorphous state. This analysis helps illuminate physical state changes that can impact the release profile and stability of the nanocarriers [134,135]. Moreover, it not only confirms whether antioxidant molecules are solubilized and dispersed evenly at the molecular level, thereby increasing the solubility and localization of the lipid matrix, but also identifies changes in the crystallinity and/or whether the order of L-NPs has changed upon the administration of a particular drug [136,137].

In recent years, molecular dynamics (MD) simulations in molecular biology and drug discovery have increased significantly since they are able to capture the behavior of biomolecules in full atomic detail and with a very fine temporal resolution by predicting the motion of each atom in biomolecule over time and exploiting a general model of the physics governing interatomic interactions [138,139]. They also enable the prediction of how biomolecules might respond to different perturbations, such as mutation, phosphorylation, protonation, the addition or removal of a ligand, etc., at the atomic level [139].

Indeed, this type of simulation has proven valuable in deciphering the functional mechanisms of biomolecules, particularly in understanding various diseases and in the design and optimization of new drugs. Among the systems suitable for molecular dynamics simulations of (bio)molecular systems is the coarse-grained force field known as the Martini force field [140].

MD simulations are often used in combination with other physicochemical characterization techniques, including X-ray crystallography, cryo-electron microscopy, nuclear magnetic resonance, and electron paramagnetic resonance.

In the design of lipid-based nanosystems, molecular dynamics has emerged as an advancement that has the capacity to postulate how drug molecules interact with lipid barriers at physiological levels. With the assistance of MD, the phospholipid membrane model has been reproduced using the Martini force field, and the trans-membrane effect of different herbal molecules with respect to their hydrophilicity has been assessed. Notably, it has been observed that the aglycon part interacts with the hydrophobic phospholipid membrane and disrupts the order of the lipophilic tails. In this regard, this strategy could provide an in-silico informative view of the distribution of drugs across biological membranes based on their solubility profiles [139].

Interestingly, this approach is also very effective in investigating the behavior of encapsulated drug molecules within the vector and toward external biological barriers. Furthermore, L-NPs were developed by solubilizing the active molecule in a lipid mixture (solid lipid or solid/liquid). The type and proportion of the constituting elements in the development of lipid carriers require careful attention. The drug loading, stability, and long-lasting effect of L-NPs are highly dependent on the selection criteria for the type of lipid(s), with the partition coefficient of the potent molecule within the lipid being the most crucial factor [141].

In the case of lipid vesicles, advancements in physical chemistry allow us to study more precisely not only the process of self-organization occurring at the interphase of the lipid/aqueous phase but also the behavior of NPs in physiological fluids. Generally, proteins are considered the target for the majority of the drugs at physiological levels, and the interaction of the DDS with bloodstream ions/proteins is a significant aspect to address during their design. The magnitude of drug–protein interactions directly affects the action and kinetic behavior of these drugs. It is important to note that there are numerous challenges in determining these interactions experimentally at nanoscale resolution within a fraction of a nanosecond. Therefore, finding the most suitable approach to predict these events has remained a major concern [142].

The atomistic/coarse-grained, Monte Carlo, and lower-resolution techniques are categorized as MD simulations, which have emerged as advanced tools of theoretical chemistry, capable of providing a deep understanding of the aforementioned interactions at both in vitro/in vivo scales. The study based on interference of the drug-containing oil globules dispersed in the micro- and nano- emulsion with the gastrointestinal fluids revealed that the variations in chain lengths and surface charge of various triglycerides affect the configuration of self-emulsifying drug delivery systems that is SEDDS [143,144]. The difference in chain lengths resulted in different kinds of droplet associations, such as random and lamellar/vesicular, that could provide a rough idea about the drug solubilization and stability within the system [145,146]. In addition, it also shows that the presence of the poly(ethylene glycol) chains at the outer surface of the particle shell prevents the nanosystem from digesting without interfering with the localized drug molecules [147,148].

It is worth nothing that, apart from blood protein–DDS interactions, the simulation studies can also illuminate the assembly process of the composing bodies based on their intermolecular interactions. Self-assembly is a vital mechanism wherein the nanostructures undergo rearrangements along with their constituting components to attain a stable unit. Images of the random mixing of lipids with active therapeutic moieties, followed by their further association with the outer aqueous phase, are captured at different nanoseconds. In other words, the mechanism of clustering the hydrophobic and aqueous parts into an organized spherical structure, that is, the nanoemulsion, can be recorded using the MD approach. This is also helpful in confirming the location of the successfully loaded molecules of interest, either in the lipid layers or in the core, as well as the types of interactions involved, namely van der Waals forces and hydrogen bonding, among others. This set of supporting information can be used to enhance the understanding of the design of L-NPs for the delivery of antioxidant molecules (ferulic acid) across various skin barriers [90].

Similarly, a study focusing on the screening of various solid lipids (different in chemical structures) to encapsulate quercetin (a well-known flavonoid) and rosemary (an essential oil) on the performance of the SLNs has also been conducted. For further understanding of the presence or absence of the essential oil, the MD simulation study has been performed. Aggregation, as the key element, was taken into consideration. For instance, in the absence of essential oil, the triglyceride lipid structures alone have shown aggregation due to a strong coherence tendency [149,150]. Theoretically, in this elucidation process, root-mean-squared deviation has been recorded, showing that the assembly system without rosemary oil was completely stable during the definite simulation period whilst, with rosemary oil, the system was fluctuating reveals no aggregation took place within the entire time frame. In order to examine the compactness and dynamic shape of systems, the value of the radius of gyration (Rg) (i.e., defined as the distribution of atoms of a protein around its axis) has been calculated [151]. In particular it reflects that the lodging of rosemary oil in the chains of the triglyceride lipids hindered the aggregation and offered reduced interfacial tension between the lipid droplets and external aqueous phase of the dispersion system [152].

Despite L-NPs, the internal structural organization of liposomes is also predictable via coarse-grained MD simulations. This tool could be helpful in the estimation of the width of the phospholipid layers, solubility, and distribution of the lipids within the droplet cores. More precisely, it can also predict how lipids are packed within the phospholipid membrane and how their presence affects the assembly pattern of other lipids. The exact time point (nanoseconds) at which the complete formation of liposomes takes place can be recorded. This approach can also describe the possible shape of liposomes, which is generally elliptical/stretched (more stable), mimicking the structures that exist within eukaryotic cells. In order to confirm stability, further calculation of the density profile across the simulation box shows how the mass of the component molecules is distributed within or outside the simulation box. It can be confirmed that the lumen structure of the vesicle lies at the center of the box containing a high density of water molecules, following the general structure of the lipid vesicles [153].

With the aid of MD, the presence of free cholesterol throughout the lipid droplet is confirmed, showing a higher concentration in the core than at the surface, a detail that was not elucidated earlier. Moreover, it can also reveal whether packed lipids undergo a series of crystallizations during preparation and storage [148].

Interestingly, the affinity of the free cholesterol molecule can be manipulated by introducing lipids with higher affinity [154]. For instance, triolein was selected and mixed with different phospholipid molecules (i.e., 1-palmitoyl-2-oleoyl-phosphatidyl- choline, POPC; 1-palmitoyl-2-oleoyl-sn-glycero-3-phospho-ethanolamine, POPE). Furthermore, by using different ratios of triolein and cholesterol, it was possible to demonstrate that free cholesterol is better miscible with triolein, allowing for the maintenance of a single-phase hydrophobic core within the lipid droplet [155].

**Figure 5:**
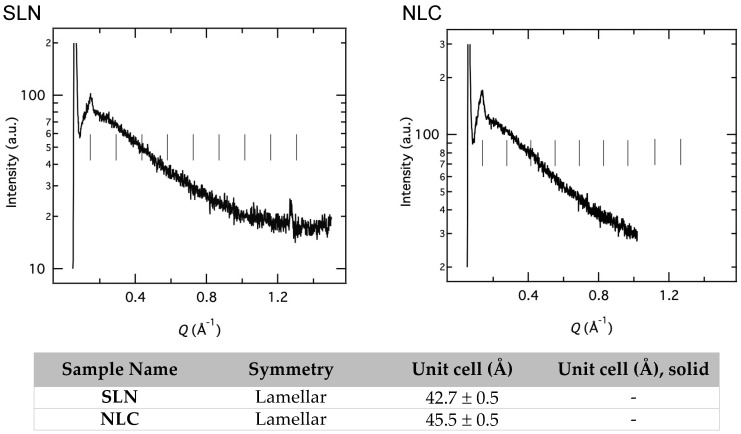
Small-angle X-ray scattering (SAXS) profiles and structural organization of SLN and NLC. The vertical black line indicates the constant position of the Bragg peaks.

Moreover, all the postulates being made with the help of MD could also be compared further by examining the small-angle X-ray scattering (SAXS) profile of the L-NPs. For instance, it has been predicted in the MD model that the insertion of drug molecules might not disturb the structural organization of L-NPs, which can later be confirmed via SAXS analysis. From the unpublished results of our research group summarized in Figure 5, SLNs and NLCs show a lamellar organization with repeated distance. However, sometimes, the shift can be seen as the influence of the presence of the loaded drug. This molecular pattern, representing the order of the phase, varies because of differences in electron densities throughout the lipid systems. If the peaks of empty and loaded lipid nanoparticles show repeated peak distance, it shows that no change in structure occurred. For example, two batches of NLCs have been designed where solid lipids (tristearin) and ellagic acid (antioxidant moiety) were kept constant, while two different liquid lipids were employed. The lamellar organization of the inner matrix was verified by the SAXS profile. It was noted that all peaks were superimposable to each other, which suggests that neither a change in liquid lipid nor the presence of a drug has changed the internal structure of the NLCs [44,153].

SAXS analysis has the ability to calculate the core radius and the reduction in shell thickness in these values that occurs when the drug is present inside. Sometimes, it is hard to corroborate these results with the DLS profile, which usually reflects a larger diameter loading of the drug. A possible reason could be that DLS is based on the diffusion coefficient, where the likelihood of a drug binding to the surface of L-NPs is high, interfering with the results. Hence, SAXS can provide a more reliable justification by calculating the size of the particles [156]. Specifically, the DLS determines the hydrodynamic diameter of the particles, while SAXS measurements are solely focused on the spherical structure, not on the solvent system. For example, DLS provides the diameter of complete PEGylated liposomes, while SAXS excludes the chains as they emerge into the solvent system [157].

Unlike, rigid nanoparticles, because liposomes are flexible vesicles, they present different SAXS profiles. In-depth, their lamellarity is always uncertain. One study was based on extruded liposomes obtained from the three different methods of preparation. It is worth highlighting that significant multi-lamellarity has been observed, even after performing serial extrusions. This unilamellar organization can be attained by insertion of PEG chains. Hence, the SAXS approach can be helpful in determining the best-fit method of preparation along with best-fit compositions [158]. Furthermore, SAXS technique is capable of observing changes on the incorporation of the charged surfactants [159,160]. Because of the presence of cationic/anionic surfactant on the surface, the liposomes resulted in a loss of the positional correlation among the adjoining bilayers, which was probably due to shifting of the charged density. It will be represented as broadband instead of congenital diffraction peaks of liposomes [86].

Apart from the elucidation of lamellarity and the point of drug localization, SAXS profiles can also shed light on the distribution of surfactants employed to stabilize the L-NPs. One study has revealed that L-NPs derived from cetyl palmitate exhibit barrel-like morphology and are surrounded by loosely associated hydrophilic surfactant (Tween 80) molecules. The hydrophilic heads of Tween 80 have a greater affinity for free water molecules, which are localized in the available space. The barrel shape is due to the stacking of platelets, each composed of a small crystalline core of cetyl palmitate, which provides a certain thickness to the *lamellae*. This information plays a vital role in delivering drug molecules to the targeted site precisely [161,162].

Apart from surfactant, it has also been found that the association of certain specific moiety, namely, α-tocopherol oxalate, along with the lipid structures, is meant to enhance structural integrity. The polar head groups of phospholipids likely have a significant affinity for inserting the moiety via dipole/electrostatic interactions, which act as driving forces to enhance the influx of water molecules; thus, a highly ordered structure was obtained [163,164].

It is interesting to note that the same cargo can elicit a non-identical response to different vectors, which can be investigated using the SAXS technique. The insertion of drug molecules can affect the internal structural organization of these nano-transporters. The SLNs have been compared with ethosomes following the incorporation of caffeic acid. However, the SAXS profile revealed that the response regarding the internal structural configuration of both carriers was completely different in relation to caffeic acid. Specifically, the addition of polyphenol to SLNs did not change the structure, whereas in ethosomes, a shift in electron density and a more ordered structural organization were observed [19]. A summary of the SAXS profiles obtained from different nanosystems containing antioxidants for wound healing is provided in Table 3.

## 6. Antioxidant Nanosystems and Their in Vivo Wound Healing Management

**Figure 6:**
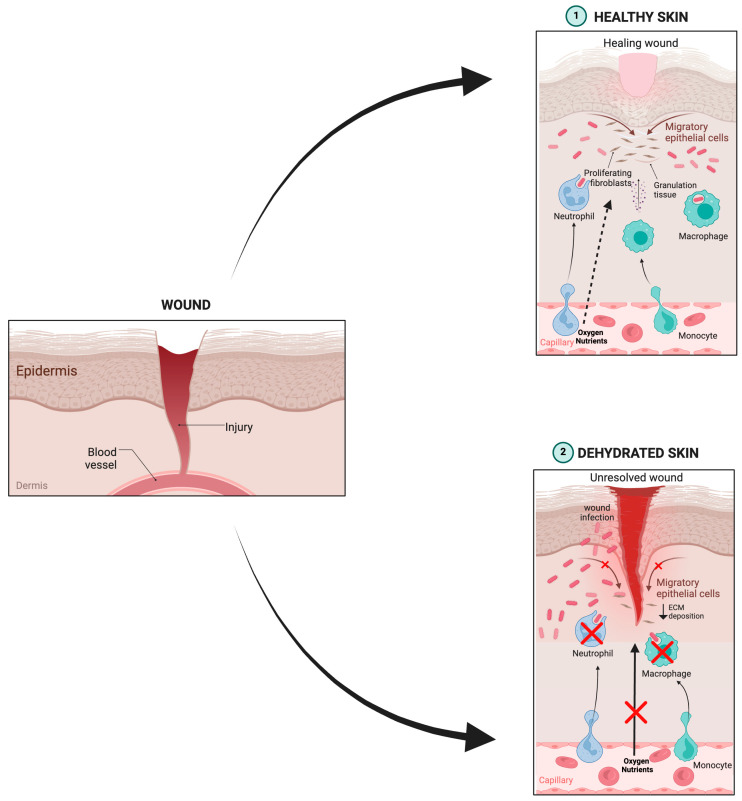
Wound healing process in skin health and dehydration conditions. (**1**) In healthy skin, increased blood irroration favors the release of oxygen and nutrients at the wound site, allowing epithelization via the migration of endothelial cells, as well as recruitment of fibroblasts that start to build new tissue (granular tissue) by releasing collagen and remodeling the extracellular matrix (ECM)**.** Moreover, the cells of the innate immune system, such as macrophages, are activated to prevent infection by removing bacteria and damaged cells. (**2**) When the skin is dehydrated, interstitial fluid pressure is reduced, leading to decreased blood circulation. As a consequence, fewer nutrients and oxygen are released in the skin tissue, compromising the migration of endothelial cells, the activation of fibroblasts, and the consequent deposition of the ECM, as well as the influx of inflammatory cells. All these events hinder skin regeneration and increase the risk of infection at the site. Original figure created with BioRender.com.

The lipid-based nanodispersions (both particulate and vesicular) have poor retention and are free-flowing over the skin surface, making them unsuitable for cutaneous administration [36]. Moreover, wound dehydration is another considerable factor that may further disturb the idyllic milieu to initiate the wound-healing process (Figure 6) [174,175]. Augmenting the residence time over the wound could be an effective approach; however, it presents numerous challenges.

The gels, serving as a secondary vehicle with semi-solid properties, are derived from natural/synthetic polymers that offer a rigid network structure and the capacity to hold small inorganic and organic particles/vesicles [176]. The information regarding different gels employed to manage wound healing is presented in Table 4.

Ideal hydrogels as wound dressings must have numerous advantages, including good mechanical strength and spreadability, along with the capacity to manage high exudate wounds; however, it is very difficult to achieve the ideal secondary vehicles [185]. Sometimes, drugs experience limited residence time and poor availability at the wound site. This issue can be addressed using film-forming spray, which can not only enhance residence time but also provide uniform drug distribution over the wound surface and facilitate their engagement in wound healing mechanism. It is a contactless mode of administration that prevents contamination/infection inside the wound. The viscoelastic polymers can be used to design film-forming sprays [186,187].

Insulin is a very strong wound-healing agent, especially in case of diabetic wounds. However, it is prone to degradation due to proteases. Also, sometimes, wounds are very painful, and no material can be applied via rubbing using the fingers. In this situation, authors proposed the encapsulation of insulin in liposomes and subsequently applying a film-forming spray [188].

**Figure 7:**
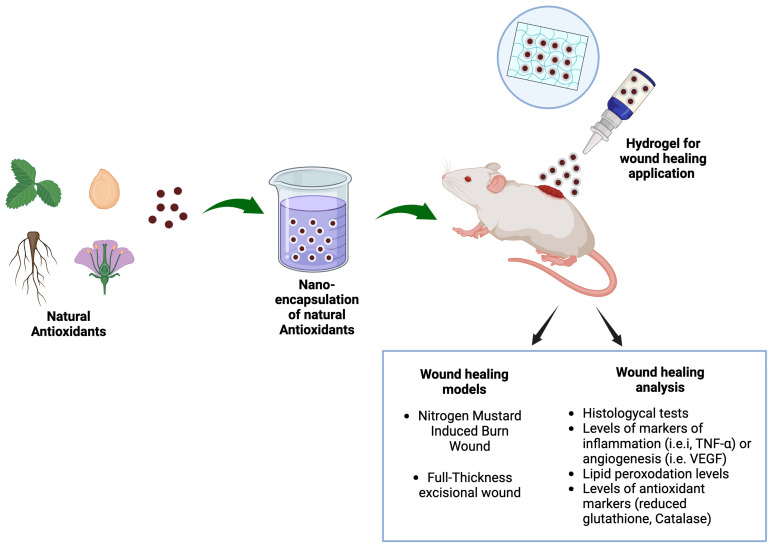
Schematic illustration of a nano-spray and a nano-particulate hydrogel derived from natural phyto-molecules for wound healing applications. Original figure created with BioRender.com.

The schematic illustration of nano-spray and nano-particulate hydrogel for wound healing applications is provided in Figure 7. Molecules with antioxidant potential can be extracted from natural sources. Later, they can be subjected to nano-encapsulation using various vectors (SLNs, NLCs, liposomes, ethosomes, transethosomes, cubosomes, etc.). In order to attain real clinical applications, these carriers can be suspended in secondary vehicles, such as nano-spray and/or hydrogel. Lastly, these final dosage forms can be evaluated for their wound healing activity using different in vivo models. The type of induced wound depends on the objective of the study, which may include simple wounds, diabetic wounds, frostbite injuries, etc.

Interestingly, the combination of the aforementioned secondary carriers (liposomes + gel + nano-spray) can also be employed in wound healing applications. Immediate action is also required to counteract the incidence of frostbite injury. In this regard, liposomes have been selected for the encapsulation of heparin sodium and ibuprofen to achieve rapid relief under extreme cold conditions, effectively aiding in cell repair and normalizing blood circulation in capillaries, thus demonstrating significant wound healing potential. In addition, for real-time application, the abovementioned liposomes were suspended in nano-spray gel, which significantly reduced the wound area [189].

## 7. Challenges and Future Prospects

Although the L-NPs are fabricated from naturally derived lipids and are biocompatible, there are still considerable challenges that need to be addressed to achieve their industrial viability, especially in the area of wound healing management. Undoubtedly, L-NPs are safe to administer, but the use of aggressive solvents for the primary solubilization of lipophilic drug molecules is a significant concern from a toxicity point of view because it is very difficult to ensure complete solvent evaporation. Solvent residues can be toxic even if the retained concentration is negligible. Therefore, efforts should be made to design a production method that minimizes energy consumption and eliminates the use of any organic solvents. An approach has been made in this direction that uses an environmentally friendly preparation method, with potential industrial feasibility, to encapsulate curcumin into SLNs for wound healing purposes [36].

Despite numerous published patents and technology transfers, SLNs and NLCs have not yet achieved significant industrial acceptance. One of the possible reasons might be a lack of complete understanding of the molecular mechanisms behind drug loading and drug release, as well as the effect of the protein corona on their behavior within the biological fluids. In addition, stability during storage remains a significant question and hurdle in achieving clinical-level success. Therefore, concrete information about the internal structural organization of L-NPs is vital before designing these systems. As previously discussed in Section 5, MD/SAXS are the best available techniques to ensure the physical and biological performance of DDS. Our previous paper emphasized the careful selection and scope of various techniques to address these challenges in the physical characterization of lipid-based nano-vectors [190]. Therefore, clinical success is plausible only after a complete understanding and characterization of L-NPs regarding physical parameters at a larger pilot scale [191].

In the area of wound healing, the transition from the bench to commercial level is difficult without considering secondary vehiculation. Therefore, in this review, we discussed the possible employment of gel or spray and gel and spray combinations that carry nanocarriers. The final product should be easily applied/reapplied to the wound without rubbing. However, the optimization of the spray is another significant challenge because the primary nanodispersion should be compatible with the secondary one. The evaluation of physical parameters is a significant hurdle, including the spray pattern and spray angle [192], balance between the viscosity and the ability of the sprayed material to reside on the wound until dried [193,194], volume delivered in one actuation, tackiness of the films formed after evaporation of the solvent, and pH of the nano-spray material [195].

## 8. Conclusions

Wound formation results from various interlinked events, including the continuous activation of macrophages and deposition of neutrophils, leading to an increase in the levels of pro-inflammatory cytokines and ROS. This phenomenon becomes more intense when wound formation takes place in diabetic patients, for whom healing is a significant concern. Therefore, a patient-friendly, concrete treatment strategy should be established. Phytochemicals with radical scavenging potential are very promising; however, they face technological challenges, including poor solubility, permeability, and stability over storage time. Lipid-based nanocarriers have received substantial attention due to their excellent properties, including deformability, elasticity, and the ability to deliver drug molecules into the deeper layers of skin tissues. However, there is a lack of understanding regarding the physical design of this lipid nano-vector. The techniques, namely, SAXS and MD, could be beneficial for fabricating optimal compositions and methods of preparation that utilize minimal energy and have greater scalability. These lipid DDS have the capacity to significantly enhance the stability of encapsulated active moieties. Despite the numerous advantages of these systems, they have not yet achieved real commercial success. Looking forward, we propose that the incorporation of these nanocarriers into secondary vehicles could open new avenues for research. However, in addition to the optimization of primary carriers, the parameters for secondary vehicles should also be thoroughly considered to facilitate the transition from bench to pilot-scale manufacturing.

## Figures and Tables

**Table 1:** Types of solid and liquid lipid blends employed in NLC formation.

Lipids		Active Molecule	EE (%) (± *s.d.*)	Remarks	Ref.
Solid	Liquid				
Myristic acid	Sunflower, olive, corn, castor, and coconut oil	Quercetin	99.90 ± *0.01*	- Increased antioxidant capacity - Controlled drug diffusion - Non-toxic to human cells	[46]
Compritol 888 ATO	Buriti oil	Amazonian fruit pulp extract	not available	- High stability and antioxidant capacity	[47]
Glyceryl dibehenate	Oleic acid	Hazelnut (*Corylus avellana*) extract	70.0 ± *0.23*	- Enzymatic transesterification reduced the oil droplet size and enhanced the effectiveness of the system.	[48]
Precirol^®^ ATO	Miglyol^®^ 812	Lutein	94.73 ± *0.03*	- Upon encapsulation, stability of lutein was improved during storage or photo-thermal treatments - The sustained release was accomplished in the presence of different surfactants	[49]
Shea butter and beeswax	Carrot seed oil	Gamma— Oryzanol	90.00 ± *not available*	- High long-term storage stability and photo-protection capability - non-irritant to the skin	[50]
GMS and capric acid	Lecithin	Propolis extract	83.29 ± *0.47* 87.21 ± *0.79*	- Increase in polyphenolic content (9-fold) and antioxidant activity (25-fold) by NLCs compared to raw extract form	[35]
Cocoa butter	Conjugated linoleic acid	Conjugated linoleic acid	98.2 ± *not available*	- Conjugated linoleic acid was protected from oxidation during the entire storage period	[51]
Precirol	Olive oil	Oleuropein	99.12 ± *0.70*	- NLCs were non-toxic to the three different lung epithelial cell lines	[52]
Palm stearin	Palm olein	β-carotene	91.20 ± *0.15*	- NLC improved solubility of the β-carotene which further enhances the targeted release and antioxidant power.	[53]
Compritol 888 ATO	Miglyol^®^ 812	Butterfly Pea Extract	72.51 ± *1.11*	- The effectiveness of NLCs also depends on the type of the solvent being used during extract formation	[54]

**Table 2:** Effect of vesicle size on active molecule permeation.

Drug	Dosage From	Mean Size (nm ± s.d.)	Penetration Potential	Ref.
Carboxyfluorescein (hydrophilic molecule)	Liposomes	70 ± 2.6 to 90 ± 2.9	Vesicles are not capable of delivering the payload into the depth of the skin layers. After drying, these vesicles may deposit in or on the SC in the form of a lipid layer.	[91]
1,1-dioctadecyl-3,3,3,3 -tetramethylindocarbo- cyanine perchlorate (lipophilic molecule)	Liposomes	58 ± 9.0	Highest deposition in viable epidermal and dermal layers of the payload can be expected	[92]
Ivabradine HCl	Transfersomes	206.7 ± 15.3	Can deliver drugs up to some extent into deeper skin layers	[93]
Not available	Not available	6–36 ± not available	The uptake can occur across both through lipidic trans-epidermal routes or aqueous pores	[94,95]
Insulin	Liposome and iontophoresis	100–400 ± not available	Drug molecules can be delivered via the trans-follicular route	[96,97]

**Table 3:** SAXS profiles of lipid-based nano-vectors carrying antioxidants for wound healing applications.

Carrier	Cargo	Composition	Remarks	Ref.
Lipid mesophases	Curcumin	Lipids extracted from the marine microalga *Nanno chloropsis* sp.	- Obtained curves confirmed non-lamellar lyotropic mesophases and ordered cubosomes - Distorted peaks represent the blend of lipids with different packing parameters that cause defects in the ordering - Curcumin has narrowed the peaks showing extended cooperativity and correlation with the neutral lipids corroborated with the NMR results which further confirmed the localization of curcumin within the bilayers.	[165]
Lipid-based nanosystem	Caffeic acid	^1^ DMPC, ^2^ DPPC and ^3^ BPL	- DPPC and BPL (with/without antioxidant) models were subjected to SAXS assessment - The hydrated phospholipids system has shown three Bragg peaks revealing lamellar phases. - These three peaks turned into one broader peak with the addition of an antioxidant. - The addition of antioxidant has disturbed the bilayers’ arrangement by increasing their bilayer thickness	[166]
Liposomes/giant vesicles/crystalline Cubic Phase	Catechin	^4^ DOPC/^5^ POPC/lipid monoolein and DOPC, chitosan	- the oxidation process of the lipids effectively leads to a change in the bilayer curvature - The introduction of chitosan-catechin provides extra stability to bilayer lipids because did not undergo any transition	[167]
Liposomes	Resveratrol	PEGylated phospholipids	- Presence of unilamellar and oligo lamellar vesicles	[168]
^6^ MAD	Curcumin	Glyceryl monooleate poloxamer 407/sodium cholate-sodium caseinate (alternate surfactants)	- A very significant variation found in internal structural organization, poloxamer 407 and/or sodium cholate-sodium caseinate resulted in cubosomes and hexosomes respectively. - Whereas, no any change due to presence of curcumin	[169,170]
	Crocin	Glyceryl monooleate, Sodium cholate Mixture of sodium cholate with sodium caseinate	- MAD with sodium cholate has shown cubosomes morphology, similarly, also empty MAD with the combination of both surfactants exhibits cubosome structure, but with crocin they turned into hexasomes.	[171]
Liposomes	Curcumin	^7^ SPC, Ascorbyl palmitate (AP)	- AP was employed to enhance liposomal physical stability: higher AP concentrations presented the most homogenous and stable liposomal dispersion - The palmitate group remarkably increased the melting point of liposomes along with the smallest possible diameter hence enhancing the physical stability.	[164,172,173]

^1^ 1,2-dimyristoyl-sn-glycero-3-phosphocholine (DMPC); ^2^ 1.2-dipalmitoyl-sn-glycero-3-phosphocholine (DPPC), ^3^ brain polar lipids (BPL); ^4^ 1,2-dioleoyl-sn-glycero-3-phosphocholine (DOPC); ^5^ 1-palmitoyl-2-oleoyl-glycero-3-phosphocholine (POPC); ^6^ MAD, Monoolein Aqueous Dispersions; ^7^ soy phosphatidylcholine (SPC).

**Table 4:** Applications of gel-containing lipid nanosystems in various disease conditions and wound healing models.

DDS	Gelling Agent	Cargo	In-Vivo Model	Remarks	Ref.
NLC	Carbopol 940	Recombinant human thrombomodulin	Streptozotocin-induced diabetic mice	- Increased granulation tissues and re-epithelialization	[177]
Liposomes	Silk Fibroin	Basic fibroblast growth factor (bFGF)	Deep second-degree scald	- The proliferation in the epidermis and dermis was accelerated - The induction of endogenous vascular endothelial growth factor (VEGF) expression initiated angiogenesis.	[178]
Hyalurosomes	Hyaluronic acid	Curcumin	Burn-wound model	- complete heal with minor or no scar within 10 days - Fast collagen formation and re-epithelization	[179]
Liposomes	Carbopol 940	Resveratrol	Wound-induced via biopsy punch in diabetic rat	- Reduction in blood glucose and elevation in glycosaminoglycans - wound closed on the 9th day. - Quicker re-epithelization, proliferation of fibroblast, formation of collagen, and reduced inflammatory cell infiltration	[180]
Liposomes	Carbopol	*Vitis vinifera* Leaf Extract	Wound infection model, and peritonitis infection model	- complete wound healing effect and tissue repair after 7 days of administration	[181]
Liposome	Glycerin and alcohol	Gallic acid	Defects were created via a metal punch in rats	- Increased fibroblast cell counts and decreased late inflammation along with increased Transforming Growth Factor β (TGF-β) expressions in the wound healing process	[182]
SLN	Carbopol 940	Fluoxetine	Wound excised with sterile toothed forceps and sharp pointed scissors	- Complete wound closure achieved within 4 weeks	[183]
SLN & niosomes	Carbopol 941	Vitamin A	Full-thickness wound model	- The improved wound healing with identical patterns in both the noisome and SLN particulate gel	[184]

## Data Availability

No new data were created.

## Abbreviations

The following abbreviations are used in this manuscript:

ROS	Reactive oxygen species
TNF	Tumor necrosis factor
IL-6	Interleukin 6
SC	Stratum corneum
FTIR	Fourier-transform infrared
DSC	Differential scanning calorimetry
NPs	Nanoparticles
L-NPs	Lipid nanoparticles
SLNs	Solid lipid nanoparticles
NLCs	Nanostructured lipid carrier
CMC	Critical Micelle concentration
MNLs	Microneedles
SEM	Scanning Electron Microscopy
TEM	Transmission Electron Microscopy
XRD	X-Ray Diffraction
MD	Molecular dynamics
DDS	Drug delivery system
SAXS	Small-angle X-ray scattering
